# HIV, disability and discrimination: making the links in international and domestic human rights law

**DOI:** 10.1186/1758-2652-12-29

**Published:** 2009-11-09

**Authors:** Richard Elliott, Leah Utyasheva, Elisse Zack

**Affiliations:** 1 Canadian HIV/AIDS Legal Network, 600 - 1240 Bay Street, Toronto, Ontario, M5R 2A7, Canada; 2 Canadian Working Group on HIV and Rehabilitation, Toronto, Canada; 3 600 - 1240 Bay Street, Toronto, Ontario, M5R 2A7, Canada

## Abstract

Stigma and discrimination constitute one of the greatest barriers to dealing effectively with the HIV epidemic, underlying a range of human rights violations and hindering access to prevention, care, treatment and support. There is some existing protection against HIV-based discrimination under international law, but the extent of states' obligations to address such discrimination has not been comprehensively addressed in an international instrument.

The United Nations Convention on the Rights of Persons with Disabilities entered into force in May 2008. As countries ratify the convention, they are required to amend national laws and policies to give greater protection to the human rights of people with disabilities, including abolishing disability-based discrimination by the state and protecting persons against such discrimination by others. The Disability Convention addresses many of the issues faced by people living with HIV (PLHIV) but does not explicitly include HIV or AIDS within its open-ended definition of "disability".

Therefore, the advent of the Disability Convention prompts us to consider the links between HIV and disability and, specifically, to consider the opportunities it and other legal mechanisms, international or domestic, may afford for advancing the human rights of PLHIV facing human rights infringements. We do so in the belief that the movement for human rights is stronger when constituencies with so many common and overlapping interests are united, and that respectful and strategic collaboration ultimately strengthens both the disability rights and the AIDS movements.

In this article, we first examine the links between HIV and disability. We then provide a brief overview of how international human rights law has treated both disability and HIV/AIDS. We note some of the different ways in which national anti-discrimination laws have reflected the links between HIV and disability, illustrated with representative examples from a number of countries. Finally, we offer some conclusions and recommendations about ways forward for collaboration between HIV and disability rights advocates in advancing human rights at the international level, including the use of the new tool that is the Disability Convention. We hope these reflections will promote further discussion across movements, ultimately to the benefit of all persons with disabilities and/or HIV.

## Introduction

Stigma and discrimination constitute one of the greatest barriers to dealing effectively with the HIV epidemic, underlying a range of human rights violations and hindering access to prevention, care, treatment and support. Some have called for the creation of a specific international human rights convention to address discrimination and other human rights violations against people living with HIV or AIDS (PLHIV). Others have felt that such an effort is impractical and unnecessary: impractical, because it can take decades to develop and negotiate a treaty through the United Nations, even where there is interest among member states; and unnecessary, because international human rights treaties have already been interpreted as prohibiting discrimination based on health status, including HIV and AIDS, which also means that discrimination that hinders the enjoyment of all other human rights protected by these treaties is also prohibited. Nonetheless, the extent of states' obligations to address discrimination on the grounds of HIV status has not been comprehensively addressed in any international treaty.

In December 2006, following intensive, years-long advocacy by disability rights activists, the UN General Assembly adopted the Convention on the Rights of Persons with Disabilities, which entered into force in May 2008 [1]. As countries ratify the convention, they are required to amend national laws and policies to give greater protection to the human rights of people with disabilities, including abolishing disability-based discrimination by the state and protecting persons against such discrimination by others. The Disability Convention addresses many of the issues faced by PLHIV, but it does not explicitly include HIV or AIDS within its open-ended definition of "disability". We recognize that whether and how HIV and/or AIDS should be conceived of as disabilities, for various legal or other purposes, is a matter of some ongoing debate among people living with HIV, people living with (pre-existing) disabilities and those advocating for human rights in these areas. There certainly are points of divergence and some tension between movements and organizations.

Therefore, the advent of the Disability Convention prompts us to consider the links between HIV and disability and, specifically, to consider the opportunities it and other legal mechanisms, international or domestic, may afford for advancing the human rights of PLHIV as a constituency facing human rights infringements. We do so with the conviction that the movement for human rights is stronger when constituencies with so many common and overlapping interests are united, and that respectful and strategic collaboration ultimately strengthens both the disability rights and the AIDS movements.

In this article, we first examine the links between HIV and disability. We then provide a brief overview of how international human rights law has treated both disability and HIV/AIDS. We note some of the different ways in which national anti-discrimination laws have reflected the links between HIV and disability, illustrated with some representative examples from a number of countries. Finally, we offer some conclusions and recommendations about ways forward for collaboration between HIV and disability rights advocates in advancing human rights at the international level, including the use of the new tool, the Disability Convention. We hope these reflections will promote further discussion across movements, ultimately to the benefit of all persons with disabilities and/or HIV.

## HIV and disability: key links

In understanding the links between HIV and (other) disabilities, it is useful first to note how conceptions of disability have developed through several stages:

The **impairment perspective **considers disability a health problem or abnormality that is situated in an individual's body or mind. This perspective is best expressed by the *medical model *which views disability in terms of disease, illness, abnormality and personal tragedy. The medical model assumes that disability is an intrinsic characteristic of individuals with disabilities. This assumption translates into practices that attempt to *fix *individuals' abnormalities and defects, which are seen as strictly personal conditions.

The **functional limitations perspective **arose from attempts to expand the medical model to include non-medical criteria of disability, especially the social and physical environment. Nonetheless, the notion that impairments are the direct cause of disability remains central to this perspective.

The **ecological perspective **... sees disability as resulting from the interaction of impairment, activity limitations and participation restrictions in a specific social or physical environment such as work, home or school [...]. There are many variations of the *social model*, but all portray disability as a social construct created by ability-oriented and ability-dominated environments ... According to the social model, even though impairment has an objective reality that is attached to the body or mind, disability has more to do with society's failure to account for the needs of persons with disabilities. The *human rights model *is a distinct subgroup of the social model. It understands disability as a social construct. The model is primarily concerned with the individual's inherent dignity as a human being (and sometimes, if at all, with the individual's medical characteristics) [2].

Appropriately, this evolution in thinking about disability has, to varying degrees in different jurisdictions, been reflected in the law, such that HIV often falls under the rubric of "disability" for at least some legal purposes -- and in particular, under anti-discrimination laws that can be used to challenge HIV-based discrimination as a form of discrimination based on disability.

The need to respond to discrimination in various forms has featured prominently in the growing calls for partnership between HIV activists and disability activists. Although in recent years the disability rights movement has made significant advances, similarly to PLHIV, people with disabilities often encounter stereotyping, discrimination and other infringement of human rights. People with disabilities are among the most marginalized in the world, and the implications of HIV infection for people with disabilities have been largely ignored. Research has identified HIV as a significant but relatively unrecognized problem among people with disabilities worldwide [3-5]. It shows higher levels of illiteracy, unemployment and poverty among people with disabilities, factors linked to vulnerability to HIV and to a greater impact of HIV infection [6].

Similarly, people with disabilities, in particular, women and young people, are at greater risk for sexual abuse or assault, elevating their risk of HIV infection [7,8]. Women, members of ethnic, sexual and other minority communities, youth, and people living in institutions are particularly at risk. It is often incorrectly assumed that people with pre-existing disabilities are not sexually active and are unlikely to use illegal drugs in ways that carry a risk of HIV infection. Thus, HIV education and other prevention efforts focused on reducing transmission through sex or drug use are rarely specifically targeted or made accessible to people with most disabilities [3]. (One notable exception is those with the medical condition of drug dependence. In a number of jurisdictions, dependence on narcotic or psychotropic drugs is recognized legally as a disability, including for purposes of protection against disability-based discrimination. Obviously, people who use such drugs, especially those who inject and those who do so frequently as a result of addiction, are particularly vulnerable to HIV infection for a host of reasons, including personal and larger structural factors that contribute to the sharing of drug-injection equipment. A significant proportion of new HIV infections globally is attributed to the epidemic of injection drug use [9]. This, then, is one group of persons with a disability that is already recognized as requiring particular attention for HIV prevention and treatment efforts, given the direct link between the disability of addiction and a high-risk behaviour. In fact, in some quarters, the call for greater, meaningful involvement of people who use drugs in the response to HIV has been expressly framed in the language of the disability rights movement's demand for the inclusion of people with disabilities [10]).

PLHIV also experience disability related to HIV. As it progresses, HIV disease can result in mental and physical conditions that impair ability. In addition, highly active antiretroviral therapy (HAART) and other treatments, while saving and prolonging the lives of PLHIV, can also cause side effects that can be disabling. In such cases, once HIV or its treatment manifests in impairment of some sort, generic legal protection against discrimination on such grounds as "disability" ought certainly to apply. However, as should be apparent from the "social model" understanding of disability, people with HIV who are asymptomatic may experience discrimination regardless of the fact that HIV does not significantly (or at all) limit their activities, and it is instead the prejudice of others which causes difficulties (e.g., in employment, housing or services), rather than HIV infection itself.

Over the past few years, there has been greater research and attention to the links between HIV and disability, growing attention by policy makers and planners, and growing awareness of the importance of ensuring access to such services. More HIV projects with a disability focus are being initiated and more resources are becoming available, although the need continues to far outstrip the response [11-14]. Consider, for example, this recent assessment by the South African National AIDS Council:

There has been a progressive improvement in the inclusion of disability in the national AIDS response, starting with minimal involvement at the beginning of 1992 by the National AIDS Coordinating Committee of South Africa (NACOSA), to full participation in the National Strategic Plan on HIV and AIDS and STI 2007-2011 (NSP 2007-2011). The NSP 2007-2011 recognises two important aspects. First, that disabled people are a group vulnerable to infection with HIV and bear the impact of AIDS severely. This recognition should lead to mobilisation of resources for disability and prioritising disabled people in the AIDS response.

Second, it recognises the causal relationship between HIV and disability. This raises the need for the disability sector and people living with HIV sector, both represented in the South African National AIDS Council (SANAC), to work collaboratively in developing programmes that respond to the causal relationship. The sectors face similar challenges, such as a struggle for self-representation and the fight for recognition of human rights. In addition, both sectors have to deal with being regarded as welfare cases, objects of medical mystery deserving of pity and ridicule." [13]

However, discussions between the disability rights movement and HIV activists reveal a gap between HIV activism and disability activism. A major factor leading to the lack of cooperation between the two movements is that both PLHIV and people with disabilities are extremely stigmatized and marginalized. Indeed, suffering from the additional burden of stigma of being seen as "disabled" has no doubt been a concern of PLHIV and AIDS rights advocates. They have often found themselves in the position of seeking to challenge discrimination based on incorrect assumptions that HIV infection *per se *renders a person unable to, for example, hold certain positions. Similarly, the intense stigma surrounding HIV, in part because of its association with sex (and in particular, disfavoured sexual minorities) and with drug-using behaviour, no doubt has created some hesitancy on the part of disability rights activists taking on HIV/AIDS as an issue of concern.

In addition, it is not a stretch to recognize that social justice advocates and movements, always struggling to secure resources to address the needs of their constituencies, will understandably be concerned that the possible conflation of HIV and disability could lead to a reduction in hard-won resources for either or both of these sectors. Similarly, in some quarters of the disability rights movement, concern has been raised that recognition of HIV as a disability could lead, under pressure from a relatively well-organized AIDS advocacy constituency, to resources being diverted away from services or advocacy for people with (other) disabilities.

However, recently there are more and more calls for greater unity between the disability rights movement and AIDS activists [15-20]. Both the disability and HIV movements could gain from increased diversity and perspective. People with disabilities are at an increased risk of contracting HIV; alliances with PLHIV and AIDS organizations can strengthen HIV education and prevention efforts to protect people with disabilities. Moreover, there are many advantages for inclusion of PLHIV as part of the disability rights movement. Where it is not already the case in domestic law, recognition of HIV and AIDS as disabilities for legal purposes may entitle PLHIV to health, employment or other benefits, as well as to the benefits of legislation protecting against discrimination, including the requirement of *reasonable accommodation *of disability (discussed below). Seeing commonalities in the stigma and discrimination experienced by both PLHIV and people with disabilities will increase tolerance and better understanding across these (overlapping) communities, and will strengthen both communities' efforts in overcoming stigma and discrimination.

Finally, working together in greater numbers will strengthen a common voice for changing public policy in ways that benefit all people, those living with HIV and those living with other disabilities. Cross-disability coalitions highlight that these issues affect an even greater portion of the population, mobilizing greater support and more attention from decision makers. For example, in the context of seeking changes to policies and programmes providing income support to people with disabilities, collaboration between HIV advocates and advocates from other disability groups not only supports exchange of knowledge on the research, policies and models that affect both groups, but also increases the potential and opportunities to inform public policy, since it engages a much broader base of people than if HIV or disability groups are working on issues alone.

## HIV and disability in the international human rights system

One such area for collaboration is in international advocacy for the human rights of people living with disabilities and PLHIV, including using the mechanisms of the United Nations to claim and defend human rights. The UN human rights system consists of numerous instruments (e.g., declarations and treaties) and a number of different offices, agencies and mechanisms for trying to ensure that governments live up to their human rights obligations.

The Universal Declaration of Human Rights sets out key human rights principles that shape the rest of international law on human rights, including the fundamental principle of non-discrimination. Numerous treaties on human rights create legally binding obligations on the governments that have ratified those treaties. Eight "core" human rights treaties are widely ratified by most of the world's countries. These protect *civil and political rights *(e.g., freedom from cruel, inhuman or degrading treatment; the right to privacy; the right to liberty; the freedom to express and to seek information, etc.) and *economic, social and cultural rights *(e.g., the right to the highest attainable standard of health; the right to equal pay for work of equal value; the right to education; the right to social insurance, etc.). They also include treaties that specifically address particular kinds of human rights abuses (e.g., torture and abduction) and forms of discrimination (e.g., racial discrimination), and the rights of particular groups, such as women, children, migrant workers -- and now, with the Disability Convention, people with disabilities. (The full text of the core human rights treaties, and their accompanying optional protocols, can be found on the website of the Office of the UN High Commissioner for Human Rights at http://www2.ohchr.org/english/law/).

Several different mechanisms exist to monitor whether and how countries are living up to their obligations under each of these treaties, and to encourage them in doing so:

▪ The **Office of the UN High Commissioner for Human Rights **(OHCHR) is the agency given the lead mandate within the UN system to protect and promote human rights, including working with governments and non-governmental organizations, undertaking investigations and studies, and being a global advocate publicly and within the UN system for human rights.

▪ The **Human Rights Council **consists of representatives from UN member states. It is the top body within the UN for dealing with human rights issues, and reports to the UN General Assembly. It meets regularly over the year, and periodically reviews each country's progress in meeting its human rights obligations through the Universal Periodic Review mechanism.

▪ The Human Rights Council can also appoint **"special rapporteurs" **and **"independent experts**", who have specific mandates to investigate and monitor the human rights performance of specific countries or to work on specific human rights issues (e.g., the right to health). These are among the "special procedures", as they are called, that can be used to place a human rights concern on the official agenda of UN member states' meetings, particularly at the Human Rights Council, but also at other bodies, such as the UN General Assembly. Special rapporteurs have also been given mandates by other UN bodies and instruments. For example, the 1993 UN Standard Rules on the Equalization of Opportunities for Persons with Disabilities created a mandate for a Special Rapporteur to monitor their implementation [21]. The Special Rapporteur reports yearly to the UN Commission for Social Development.

▪ Finally, each of the core human rights treaties is overseen by a **committee **that consists of independent experts that regularly review countries' progress under the treaties. In some cases, depending on the wording and ratification of one or more Optional Protocols to these treaties, these committees can receive complaints from individuals or groups about specific human rights violations by government, and can issue findings and recommendations to governments to remedy the situation. In the case of the Disability Convention, the treaty itself creates the Committee on the Rights of Persons with Disabilities, to which all states parties to the convention must submit regular reports. The Optional Protocol to the Disability Convention empowers the committee to examine and "judge" complaints, against those states that have ratified the protocol, from individuals and groups of individuals alleging violations of the convention [22]. The Optional Protocol also empowers the committee to initiate inquiries *vis-à-vis *a state party on the basis of reliable information indicating "grave or systematic violations" of the convention, although such an inquiry requires the consent of the state party, and any state that ratifies the Optional Protocol may "opt out" of this inquiry procedure.

These different parts of the UN human rights system can be used to protect and promote the human rights of PLHIV and people with disabilities, including the rights recognized and protected by the Disability Convention.

### International human rights law and disability: what room for PLHIV?

There is no one universally accepted definition of "disability" in international human rights law. A number of different definitions are commonly used. While none explicitly recognizes HIV or AIDS as disabilities, a number of them could or have been interpreted as including HIV and AIDS.

Consider, for example, the 1993 UN General Assembly declaration setting out the UN Standard Rules on the Equalization of Opportunities for Persons with Disabilities, which became the leading instrument within the UN system addressing the human rights of people with disabilities, including through the work of the Special Rapporteur. The Standard Rules also helped inform national legislation in some UN member states. In defining disability, the Standard Rules simply state: "People may be disabled by physical, intellectual, or sensory impairment, medical conditions or mental illness."[21] However, these Standard Rules are not clearly legally binding on governments in the way a treaty is binding. What recognition, then, is there of the rights of people with disabilities within more robust elements of international law?

Within the regional human rights systems, the definition of "disability" in the 1999 Inter-American Convention on the Elimination of all Forms of Discrimination against Persons with Disabilities (Article I) is certainly broad enough to encompass HIV/AIDS: "a physical, mental, or sensory impairment, whether permanent or temporary, that limits the capacity to perform one or more essential activities of daily life, and which can be caused or aggravated by the economic and social environment."[23]

At the global level, the UN's International Covenant on Economic, Social and Cultural Rights (ICESCR) is one of the core human rights treaties [24]. It does not refer explicitly to persons with disabilities. However, the treaty itself declares, in Article 2(2), that the rights it sets out are to be "exercised without discrimination of any kind" based on certain grounds explicitly listed in the treaty or based on "other status". The UN Committee on Economic, Social and Cultural Rights, the expert committee tasked with interpreting the treaty and monitoring states' progress in its implementation, has adopted a number of General Comments, which serve as authoritative expert interpretations of the treaty's provisions. General Comment No. 5 states clearly (in Paragraph 5) the committee's expert opinion that the prohibition on discrimination based on "other status" clearly includes discrimination on the grounds of disability [25]. The committee also makes clear (in Paragraph 15) that "disability-based discrimination" includes any distinction based on disability, or the denial of *reasonable accommodation *of a disability, which limits or denies any of a person's economic, social or cultural rights set out in the ICESCR. It recognises, in language that could just as easily be applied specifically to the experience of many PLHIV, that

... through neglect, ignorance, prejudice and false assumptions, as well as through exclusion, distinction or separation, persons with disabilities have very often been prevented from exercising their economic, social or cultural rights on an equal basis with persons without disabilities. The effects of disability-based discrimination have been particularly severe in the fields of education, employment, housing, transport, cultural life, and access to public places and services [25]

Indeed, in General Comment 14, in which the same committee elaborates on the right to the highest attainable standard of health under Article 12 of the ICESCR, it has also explicitly noted that the right to health includes *accessibility *(including for persons with disabilities and persons living with HIV/AIDS) of health facilities, services and information [26]. The committee has recommended that "comprehensive anti-discrimination legislation in relation to disability would seem to be indispensable in virtually all States parties" [26].

In addition to the ICESCR, the Convention on the Rights of the Child, another of the "core" human rights treaties, expressly prohibits any discrimination in respect of the enjoyment of convention rights on the ground of disability, and explicitly mentions the rights of children with disabilities (in Articles 2 and 23) [27]. Until the adoption of the Disability Convention, it was the only core human rights treaty to mention disability explicitly.

In December 2006, the UN General Assembly adopted the Disability Convention. (It is worth noting that it is the first UN human rights treaty to mention explicitly, in Article 25, the right to sexual and reproductive health - a right of obvious relevance for HIV prevention, treatment, care and support among persons with disabilities and of considerable significance to people living with HIV.) The convention does not include a definition of "disability" or "persons with disabilities"; nor does it expressly mention HIV or AIDS.

However, reflecting the ecological model of understanding disability, the convention's preamble recognises that "disability is an evolving concept and that disability results from the interaction between persons with impairments and attitudinal and environmental barriers that hinders their full and effective participation in society on an equal basis with others". Article 1 of the convention further states: "Persons with disabilities include those who have long term physical, mental, intellectual or sensory impairments which in interaction with various barriers may hinder their full and effective participation in society on an equal basis with others."

Discrimination on the basis of disability is defined in Article 2 of the convention as "any distinction, exclusion or restriction on the basis of disability which has the purpose or effect of impairing or nullifying the recognition, enjoyment or exercise, on an equal basis with others, of all human rights and fundamental freedoms in the political, economic, social, cultural, civil or any other field".

Before the advent of the Disability Convention, there was no comprehensive treaty in international law addressing the human rights of persons with disabilities. While it is commonly said that the Disability Convention "does not create new rights", it nonetheless represents a major breakthrough in articulating what the existing human rights obligations in international law require of states in order to ensure the equal enjoyment of all human rights by all persons with disabilities. It covers many areas in which persons with disabilities have faced discrimination, including access to justice, participation in political and public life, education, employment, freedom from torture, exploitation and violence, and freedom of movement.

The convention identifies areas where adaptations have to be made so that persons with disabilities can exercise their rights and areas where the protection of their rights must be reinforced because those rights have been routinely violated. Under the convention, as is the case with many national laws, discrimination based on disability includes the denial of "reasonable accommodation", defined in Article 2 as:

necessary and appropriate modification and adjustments not imposing a disproportionate or undue burden, where needed in a particular case, to ensure to persons with disabilities the enjoyment or exercise on an equal basis with others of all human rights and fundamental freedoms.

Reasonable accommodation could, for example, oblige an employer to provide a desk that can accommodate a wheelchair, allow a flexible work schedule for medical purposes, modify instructions or reference manuals, or provide equipment that will enable a person with a visual or hearing impairment do his or her work.

### International human rights law and HIV

There are several *non-binding *international instruments (e.g., declarations and recommendations) that explicitly address discrimination on the basis of HIV. Major international human rights treaties have been interpreted to include HIV as a ground on which discrimination is prohibited.

For example, the UN Committee on Economic, Social and Cultural Rights has confirmed that the term "other status" in the ICESCR includes "health status", which in turn includes HIV/AIDS (just as the term has also been interpreted to include disability) [26]. In its General Comment on HIV and children's rights, the Committee on the Rights of the Child has affirmed that the general principle of non-discrimination in the Convention on the Rights of the Child must be one of the "guiding themes" in responding to HIV/AIDS as it affects children, which must include not discriminating against children living with HIV/AIDS (Paragraphs 5 and 7) [28].

There is, however, no one international binding document expressly prohibiting discrimination on the basis of HIV or AIDS; the protection is entirely contingent upon this inclusive interpretation. In addition, as these broader human rights treaties are drafted at a level of generality, they do not offer the specific guidance of how the obligations might apply specifically in the context of HIV/AIDS - something that, as noted, the Disability Convention now very importantly elucidates in the context of disability.

Such HIV-specific guidance to states regarding their human rights obligations is to be found in the International Guidelines on HIV/AIDS and Human Rights. First issued in 1998 by the Joint United Nations Programme on HIV and AIDS (UNAIDS) and the Office of the UN High Commissioner for Human Rights (OHCHR), and subsequently revised in 2006 to reflect new developments, the stated purpose of the International Guidelines (Paragraph 3) is "to assist States in translating international human rights norms into practical observance in the context of HIV" [29]. The International Guidelines emphasize that states should enact or strengthen anti-discrimination and other protective laws that protect vulnerable groups, people living with HIV/AIDS and people with disabilities from discrimination in both the public and private sectors, as well as provide for speedy and effective administrative and civil remedies for discrimination.

The International Guidelines have been supported repeatedly by UN member states through resolutions adopted at the UN Commission on Human Rights (the predecessor body to the current UN Human Rights Council) [30-34]. In affirming the International Guidelines, the UN Commission on Human Rights has repeatedly urged states to take all necessary measures to eliminate stigmatization and discrimination against those infected and affected by HIV/AIDS. The Commission on Human Rights has confirmed that discrimination on the basis of AIDS or HIV status, actual or presumed, is prohibited by existing international human rights standards, and that the term "or other status" in non-discrimination provisions in international human rights texts should be interpreted to cover health status, including HIV/AIDS [31-36].

At the level of technical experts within the UN system, it is recognized that HIV should be considered to fall within the definition of "disability" for at least the purposes of anti-discrimination law. For example, in its 1996 statement before the UN Commission on Human Rights, UNAIDS recommended that HIV/AIDS should be considered a disability in light of the discrimination that occurs because of HIV/AIDS, and in light of the legal protection needed to guard against that discrimination:

"The so-called disabling feature either does not disable at all, but is perceived as disabling; or it may disable somewhat, but could be addressed with reasonable accommodation. The main thing is that there is no justification for differential treatment. The disabilities consequences of asymptomatic HIV is that often people living with HIV, as well as those suspected of being HIV positive, are very often discriminated against because they are wrongly perceived as being unable to perform; they are wrongly perceived as being a threat to public health; or they are perceived as being, or indeed are, a member of some group already suffering discrimination. Thus, if they are not actually disabled by HIV-related conditions, they are often disabled by the discriminatory treatment they receive because of their HIV status. The result is that they are denied the possibility of being productive, self-reliant, full and equal members of society ... Thus, the clinical, social and cultural elements of HIV/AIDS, including the impairment which can result from it and the ignorance, discrimination and stigma which surround it, confirm that it is appropriate to consider HIV/AIDS as a disability for purposes of protection against discrimination."[37]

UNAIDS also stated that in order to fully protect the people who face discrimination because of actual or perceived notions regarding their abilities due to their health status, definitions of disability should move beyond functional limitations to cover medical conditions, such as HIV/AIDS [37].

This reflects the recognition that disability is an evolving concept, a foundational premise later articulated in the Disability Convention. More recently, the Handbook for Parliamentarians on HIV and AIDS, updated in 2007 by UNDP, UNAIDS and the Inter-Parliamentary Union, recommended, as one of the components of anti-discrimination legislation, that states provide protection against discrimination on the grounds of disability, widely defined to include AIDS [38]. In March 2008, the Africa Campaign on Disability and HIV/AIDS adopted the Kampala Declaration on Disability and HIV/AIDS, which calls for HIV/AIDS to be included as "a cause of disability" [20].

Finally, in the UN General Assembly, member states have decried discrimination against PLHIV as contrary to human rights principles, although they have not cited specific treaty provisions, nor have they been willing to explicitly adopt or endorse *holus bolus *the more detailed expert commentary accompanying the International Guidelines. In 2001, the UN General Assembly adopted the Declaration of Commitment on HIV/AIDS, in which states committed to enacting, strengthening and enforcing legislation, regulations and other measures to eliminate all forms of discrimination against people living with HIV/AIDS and members of vulnerable groups [39]. They reaffirmed this commitment in the Political Declaration on HIV/AIDS, adopted in 2006 [40].

## National anti-discrimination laws: disability and HIV

There are several ways in which countries have dealt with HIV-based discrimination in national legislation, reflecting different approaches to understanding the relationship between HIV and (other) kinds of disability when it comes to protecting against discrimination.

*General anti-discrimination laws *prohibit discrimination against classes of persons, based upon such factors as race, gender and religion, and sometimes based on such grounds as "health status" and/or "disability" (or sometimes "handicap"). These latter terms could be interpreted as including HIV and/or AIDS. Not many countries explicitly include HIV or AIDS as standalone grounds on which discrimination is prohibited. In some cases, the law may only include (or be interpreted to include) AIDS or opportunistic infections and other health conditions related to HIV infection.

Some countries pass *HIV-specific laws*, which often address a wide range of HIV-related legal issues, and usually include provisions that prohibit discrimination based on HIV status and/or AIDS diagnosis. In some cases, this is the only protection in a country's law against such discrimination. In other cases, the section on discrimination in the country's "AIDS law" may clarify, supplement or reinforce protection already found in other anti-discrimination laws, where these exist and include HIV or AIDS in one way or another, such as under the rubric of protection against discrimination based on disability.

In some countries where the law prohibits discrimination based on "disability" (or, in some cases, "handicap"), HIV-positive status and/or AIDS diagnosis is included *per se *within this term. For example, in a number of countries (e.g., a number of common law jurisdictions, such as Australia, Canada, the United Kingdom, the United States, Ireland and New Zealand), anti-discrimination laws prohibiting discrimination based on disability include *asymptomatic HIV infection *within the ambit of the term "disability", either by explicit statutory language or as a result of statutory interpretation by courts and tribunals.

In some cases, national anti-discrimination law covers "disability which exists at present, previously existed but no longer exists, or which may exist in the future or which is imputed to a person", and also covers disability which is "suspected or assumed or believed to exist or to have existed" [41]. In many European countries, there are general prohibitions on discrimination where disability is mentioned but not defined. In the EU Framework Directive, the issue of disability definition was left to the member states in order to give them the opportunity to use their own national legislative definitions [42].

In other cases, obtaining legal protection against disability-based discrimination depends on proving in a given instance that a person's ability to perform certain life activities, such as work or education, is limited by some condition. Some laws use broad definitions covering minor disabilities, while others use detailed definitions that limit coverage to people with substantial disabilities. To restrict the scope of protection too much could mean excluding people who suffer from episodic illness or disability (of obvious relevance to people with HIV and others whose functional limitations may vary considerably over time). Too narrow an approach could also mean the law fails to address the discrimination that manifests from stereotypes, prejudice and general social stigma, such as that faced by PLHIV and those with other disabilities, or discrimination based on perceived disability - both of which can in themselves be disabling by limiting a person's participation, for example, in the workforce or school. In other words, such an approach would reflect more the "impairment perspective" or the "functional limitations perspective", rather than the more modern "ecological perspective" or "social model" of understanding disability and the barriers to equal enjoyment of human rights for persons with disabilities. In the remainder of this section, we provide some examples of different approaches taken to addressing HIV/AIDS-based discrimination in a number of jurisdictions from different regions.

### Defining or interpreting "disability" to include HIV/AIDS

One approach, such as that taken in Australia's federal statute on disability-based discrimination, is to include in the definition of disability broad language referring to disease or illness, such as the following: "the presence in the body of organisms causing disease or illness; or the presence in the body of organisms capable of causing disease or illness" [43]. New Zealand's general anti-discrimination statute uses the same formulation (s. 21(2))[41], as does Ireland's law on discrimination in employment (s. 2(1)) [44], both of which define "disability" similarly as including "the presence in the body of organisms causing, or likely to cause, chronic disease or illness".

In other instances, legislation on disability discrimination singles out HIV/AIDS for explicit mention as being included within the ambit of "disability". For example, Hong Kong's Disability Discrimination Ordinance (s. 2) uses the same formulation as Australia's federal statute, but supplements the generic reference to disease or illness with an additional, explicit statement (s. 61(2)) that persons who are HIV positive or have AIDS are protected by the ordinance [45]. Similarly, the United Kingdom's existing legislation on discrimination (and specifically, disability-based discrimination) has been amended to include HIV and/or AIDS explicitly within the definition of the term "disability". Section 1 of the Disability Discrimination Act of 1995 (DDA) defines a disabled person as someone who "has a physical or mental impairment which has a substantial and long-term adverse effect on his ability to carry out normal day-to-day activities" [46]. In 2005, amendments to the DDA extended discrimination protection to apply to person living with HIV, as of the moment of diagnosis: "A person who has cancer, HIV infection or multiple sclerosis is to be deemed to have a disability, and hence to be a disabled person."[47]

In other instances, legislation addressing discrimination does not clearly refer to illness or disease, or an agent thereof, in the definition of disability, but nonetheless recognizes a link between health and disability. For example, in 1990, following protest over discrimination against PLHIV, France passed a law prohibiting discrimination on the grounds of "health status or handicap" in the form of a number of amendments to the Penal Code (criminalizing certain acts of discrimination) and the Labour Code, adding these two grounds simultaneously to existing prohibitions on discrimination on such grounds as race, nationality, religion, sexual orientation or marital status [48]. Further legislation in 2005 adopted additional provisions aimed at strengthening the equality rights and social participation of people with disabilities; that law (Art. L 114) set out a new definition of "handicap" in the Family and Social Action Code as follows:

For purposes of this Act, "handicap" includes any activity limitation or restriction on participation in social life experienced by a person in his or her environment as a result of a substantial alteration, either ongoing or of a fixed term, of one or more senses or physical, mental, cognitive or psychic functions, of a multiple handicap or of a disabling health problem [49].

The use of the expression, "health status or handicap", in the French statute, and the legislative history relating to HIV, establishes that HIV/AIDS is covered. It is also worth noting this example of an instance in which AIDS activists succeeded in obtaining the expansion of legislative protection against discrimination in a manner that benefits a very broad range of people with other disabilities, with a definition that extends to any medical condition or impairment.

In other jurisdictions, anti-discrimination legislation may not explicitly refer to HIV/AIDS, and may not include in the definition any reference to illness or disease or even such a term as "health status", yet courts and tribunals have repeatedly interpreted such terms as "handicap" or "disability" as including HIV and/or AIDS for purposes of protecting against discrimination. For example, none of the anti-discrimination statutes in force in any of Canada's 13 jurisdictions (one federal, 10 provincial and three territorial) make explicit reference to HIV. Yet human rights commissions, tribunals and courts at all levels have had little difficulty in finding that such statutes' prohibition on disability-based discrimination includes discrimination based on actual or perceived HIV or AIDS status [50]. The Supreme Court of Canada, reflecting the "social model" understanding of disability and related discrimination, has observed that:

Whatever the wording of definitions used in human rights legislation, Canadian courts tend to consider not only the objective basis for certain exclusionary practices (i.e. the actual existence of functional limitations), but also the subjective and erroneous perceptions regarding the existence of such limitations. Thus, tribunals and courts have recognized that even though they do not result in functional limitations, various ailments such as congenital physical malformations, asthma, speech impediments, obesity, acne and, more recently, being HIV positive, constitute grounds of discrimination ... [51]

Similarly, in the United States, the Americans with Disabilities Act of 1990 (ADA) does not mention HIV or AIDS, or any other disabling conditions directly. Under the ADA, "the term 'disability' means, with respect to an individual (a) a physical or mental impairment that substantially limits one or more of the major life activities of such individual; (b) a record of such an impairment; or (c) being regarded as having such impairment."[52] But the US Supreme Court has confirmed that HIV and AIDS both qualify as "disabilities" under the ADA [53].

### Explicit protection against discrimination based on HIV/AIDS status

A different approach treats HIV as distinct, although in some ways connected to, disability. We suggest the approach apparent in the examples below arises largely out of a desire to avoid reinforcing a perception that PLHIV are, simply by virtue of their HIV infection, limited in functioning (e.g., in employment) - in other words, to avoid subjecting PLHIV to the further stigmatization and discrimination attached to disability.

For example, early government policy in South Africa acknowledged the similarities in discrimination experienced by PLHIV and people with disabilities, but drew the line at characterizing HIV infection *per se *as a disability:

People who are HIV positive suffer from social discrimination similar to that experienced by people with disabilities. This does not, however, imply that they are necessarily disabled. For the purpose of the *Integrated National Disability Strategy *therefore, they are not included in the definition of disability, except where symptoms, such as prolonged fatigue, interfere with their normal functioning [54].

South Africa is a developing country that has not adopted the approach of enacting an omnibus "AIDS law". Instead it has addressed HIV as relevant in a variety of other existing legal instruments and regimes, including those dealing with discrimination of various forms. In doing so, while government policy recognizes the links between HIV and disability (including recognizing that HIV disease may cause disabilities) [13], it has also maintained a distinction in anti-discrimination law between the two, rather than simply subsuming HIV under the broader category of "disability".

For example, the Employment Equity Act in South Africa prohibits "unfair discrimination" in employment on a variety of grounds. It explicitly lists both "disability" and "HIV status" as distinct grounds on which discrimination in employment is prohibited [55]. (The EEA's provisions regarding HIV are further fleshed out in the Code of Good Practice on Key Aspects of HIV/AIDS and Employment, issued by the Minister of Labour in 2000; the Act itself declares that it is to be interpreted not only in compliance with the national Constitution, but also with such codes of good practice.) The EEA was the first law in South Africa to address directly the issue of discrimination in employment on the basis of HIV status. (Note that, in recognition of the discriminatory motivation behind demands for employment-related HIV testing, and the potential for discrimination that may follow a positive test result, the EEA also contains a general prohibition, with some exceptions, on medical testing of an employee and explicitly prohibits HIV testing unless this is found to be justifiable by the Labour Court: s. 7.) The EEA applies to most employers, workers and job applicants in South Africa (with the exception of certain named defence and intelligence bodies). The definition of disability in the Act (s. 1) is as follows: "'People with disabilities' means people who have a long-term or recurring physical or mental impairment which substantially limits their prospects of entry into, or advancement in, employment."

Another example, also from sub-Saharan Africa, illustrates an even more explicit (legal) distinction being drawn between HIV and disability, in which the former is expressly excluded from the category of the latter. Mauritius, which has been recognized as having one of the world's more progressive omnibus "AIDS laws", has expressly declared that HIV and AIDS are *not *disabilities, while nonetheless explicitly prohibiting discrimination based on actual or presumed HIV infection or AIDS diagnosis, and also allowing that a person with HIV or AIDS might nonetheless, in the right circumstances, be entitled to a disability pension benefit. Article 3 of the Mauritian law provides as follows:

(1) Any person who is HIV-positive or has AIDS shall not be considered as having a disability or incapacity by virtue of any enactment and his status or presumed status shall not be used as a ground to discriminate against that person.

(2) Subsection (1) shall not affect the operation of a pension law if that law provides for a benefit accruing to a person according to the degree of disability which entitles him to such benefit [56].

In other jurisdictions, there may be a similarly narrow "functional" approach to recognizing HIV/AIDS as a disability while also providing explicit protection against HIV/AIDS-based discrimination (albeit often with exceptions to that protection which are unjustifiably broad), even where the law contains little in the way of a more general protection against disability-based discrimination. This is the case, for example, in a number of countries previously forming part of the Soviet Union, where there are specific HIV laws which including provisions prohibiting discrimination against people with HIV.

In the Russian Federation, for example, there are specific articles that prohibit employment discrimination, denial of medical care and other limitations of rights and interests of people living with HIV, including their and their families' housing rights [57]. However, a person with HIV is considered "disabled" under the law only if HIV or AIDS causes physical impairments or full or partial loss of employment abilities. A separate Russian statute defines "persons with disabilities" as "persons with health impairments caused by disease, traumas or other reasons, which have long lasting effect on bodily functions and lead to limitations of activity and necessitate social protection" [58]. The law provides people with disabilities with employment benefits and establishes a quota system, reserving a certain number of places for persons with disabilities in the training and employment programmes in all public and private entities of more than 20 staff members, but does not contain any specific anti-discrimination provisions.

### Key point: ensure protection against discrimination based on HIV/AIDS status

UNAIDS suggests that the inclusion of HIV in national disability laws has been one of the most effective means by which to address discrimination based on HIV status or AIDS [37], although as the review above indicates, it is not the only way in which to tackle HIV-related discrimination. The overriding objective needs to be to ensure effective and adequate protection for people with HIV and people with (other) disabilities against discrimination and access to the necessary means of challenging and remedying such discrimination when it occurs. Commenting in particular on the situation of discrimination in employment or services, UNAIDS notes that the most effective laws have the following elements:

• They address people with HIV, including the full spectrum, from asymptomatic infection to AIDS.

• They include people merely perceived as having HIV or AIDS.

• They prohibit employers and providers of services from refusing to hire, from refusing to promote, from firing, and from denying services because a person is HIV positive or may become sick in the future or may cause an increase in health care or insurance costs.

• They are applicable to a broad range of public and private sector employers and service providers.

• They require that a person be qualified for the job and be well enough to perform the job adequately, but also that employers provide reasonable accommodations to facilitate ability to perform [37].

Similarly, as the World Bank has observed: "The most comprehensive laws and policies extend protection to actual, perceived, or suspected HIV status to cover those who are discriminated against due to actual HIV-infection or the perception that they are infected because of proximity to others perceived to be infected (e.g., family members) or association with groups stereotypically linked with HIV infection" [59].

In our view, a more nuanced understanding of the links between HIV/AIDS and other forms of disability, and a more modern "social model" of understanding disability, lead to the conclusion that, at least for purposes of anti-discrimination law, HIV and AIDS are legitimately viewed as a "disability" - and that where it exists, protection against discrimination based on disability should extend to protecting against discrimination against PLHIV.

This is not to deny that other approaches may also be pursued to achieve this objective, and in some instances, these may even be complementary, thereby enhancing legal protections against HIV/AIDS-related discrimination. But in at least some jurisdictions, a broader understanding of "disability" within or under existing legislation offers a key means of addressing discrimination against PLHIV and should be utilized to maximum benefit.

On the international level, a similar conclusion follows with respect to the Disability Convention. Therefore, in the next, concluding section, we return to examining how the convention, the most recent addition to the core human rights treaties, may be used by human rights advocates to strengthen protection against discrimination based on HIV or AIDS status in international law.

## Conclusion

In one its earliest judgments under South Africa's new post-apartheid constitution, the country's Constitutional Court considered whether South African Airways had violated the constitutional prohibition on "unfair discrimination" by refusing to hire an HIV-positive person as a cabin attendant on the claimed basis that HIV rendered him unable to perform the essential functions of the job. The South African Constitution does not explicitly refer to HIV, but does explicitly refer to disability. In its judgement, the Constitutional Court explicitly declined to address whether the discrimination was based on disability or whether people with HIV ought *not *to be regarded as having a disability (as argued by the AIDS Law Project as *amicus curiae*).

Nonetheless, in concluding that the employer airline had engaged in unfair discrimination, the court stressed the following underlying point of common relevance to the struggle for equality of both people with HIV and people with (other) disabilities recognized as such:

At the heart of the prohibition of unfair discrimination is the recognition that under our Constitution all human beings, regardless of their position in society, must be accorded equal dignity. That dignity is impaired when a person is unfairly discriminated against. The determining factor regarding the unfairness of the discrimination is its impact on the person discriminated against. Relevant considerations in this regard include the position of the victim of the discrimination in society, the purpose sought to be achieved by the discrimination, the extent to which the rights or interests of the victim of the discrimination have been affected and whether the discrimination has impaired the human dignity of the victim [60].

Discrimination based on HIV-positive status and discrimination based on disability, whether or not HIV is perceived or treated legally as a disability, share these basic hallmarks of unfair treatment.

In this article, from the perspective that HIV can and should be recognized as a disability for at least some purposes, including anti-discrimination law, we have noted some aspects of the multi-faceted relationship between HIV and other forms of disability, as well as the ways in which international and (some) national laws deal with discrimination related to both HIV and disability. In so doing, we hope to provide a basis for considering the implications, challenges and opportunities of recognizing HIV as a disability, including seeking such an explicit interpretation of the UN's Disability Convention.

Through consultation and discussion with AIDS advocates, disability rights advocates, people living with HIV and with other disabilities, and other interested actors, the ultimate goal is to identify and develop potential strategies for achieving better protection and promotion of the rights of people living with HIV and with disabilities. In our view, just as national law on disability-based discrimination should extend to address HIV/AIDS-based discrimination, so too should the provisions of the Disability Convention apply in the context of HIV/AIDS and extend to protect the rights of PLHIV.

As noted above, unlike the situation (now) with disability, there is no binding international law instrument dealing explicitly and directly with HIV and human rights. The term, "other status", in at least some international human rights treaties has been interpreted to include HIV. However, this remains at the level of "soft law" interpretations (e.g., by expert committees in the UN system) or recommendations (e.g., by UNAIDS and OHCHR in the International Guidelines on HIV/AIDS and Human Rights). The extent to which such treaty obligations get reflected in national legislation in ways that extend these human rights to PLHIV is, therefore, heavily dependent on the willingness of domestic legislators or national-level courts and tribunals to adopt such an interpretation.

There is no guarantee that protection against discrimination on the ground of "health status", as articulated in international treaties, will be interpreted broadly, so as to include HIV/AIDS, in national legislation and by national courts or tribunals. Therefore, the protection against violations of the rights of PLHIV that is ostensibly afforded by such existing treaties may also depend on the capacity of domestic advocates to make use of the relevant UN human rights mechanisms to press for country-level change to reflect this common interpretation.

The Disability Convention offers the prospect of a binding treaty spelling out specific obligations of states parties that would be of benefit to PLHIV - *if *HIV and AIDS are understood as disabilities falling under the rubric of the convention. As has already been noted, the term "disability" is not defined in the Disability Convention; it does not, therefore, offer the explicit extension of the protections of international human rights law to PLHIV. However, if it is made clear that the term "disability", in a legally binding international treaty such as the Disability Convention, includes HIV/AIDS, then it will be obligatory for countries that ratify the treaty to ensure that national legislation on the rights of persons with disabilities, including protection against discrimination, extends to protect PLHIV, either explicitly or by interpretation.

As has been widely recognized, the Disability Convention does not create new rights. However, it does flesh out, in considerably greater detail than has ever been the case, states' obligations under other human rights treaties in the context of disability, making it clear that people with disabilities are entitled to the full enjoyment of those rights set out elsewhere in international law. (Very practically speaking, this could strengthen efforts to ensure access to social and other services and supports available to persons with disabilities, services that can be critically important to PLHIV.) This is the chief "added value" of the Disability Convention in the international human rights framework and should not be underestimated. To the extent that this much more detailed treaty is clearly recognized from the outset as applying to HIV/AIDS, then it only strengthens the clear application of international human rights treaties to protect the full enjoyment of a wide range of human rights by PLHIV. As a preliminary list, we therefore suggest a number of possible areas for action by advocates for using the Disability Convention as a new tool for advancing the human rights of PLHIV.

First, the Disability Convention (Article 33) obligates states parties to create one or more focal points domestically to be responsible for implementation of the convention. Those focal points should be engaged to think about the HIV-relevant dimensions of the convention, such as in gathering data about HIV-related discrimination, and formulating policy to address disability-related discrimination in a way that includes HIV and AIDS as disabilities.

Second, the Disability Convention (Article 38) contemplates the engagement of UN specialized agencies in the implementation of the convention. This provides an opportunity for an agency, such as UNAIDS, to engage with states parties, the Committee of Experts, the OHCHR and even national focal points to ensure attention to the links between HIV and disability are considered in relation to the convention. This could include such initiatives as:

• Providing input on questions that the committee should put to states parties for their regular reporting;

• Participating in the preparation of a general recommendation on HIV/AIDS and the convention;

• Joint efforts by UNAIDS and OHCHR documenting and analyzing disability-based discrimination against PLHIV, as well as the ways in which the infringement of human rights of people with pre-existing disabilities contribute to HIV vulnerability and barriers to care; and

• Advising on the development of national law or policy on disability (e.g., ensuring attention to HIV/AIDS and the disabilities experienced by PLHIV) and law or policy related to HIV/AIDS (e.g., ensuring attention to disability and its links to HIV, as well as referencing the Disability Convention as an international treaty imposing relevant obligations on the state in question).

Third, the Committee on the Rights of Persons with Disabilities, the committee of independent experts established under the convention (Article 34), will, in the coming years, elaborate on what the Disability Convention requires of states in various areas. (First constituted in November 2008 with an initial complement of 12 members, the committee will expand to 18 independent experts once 60 states have ratified the convention.) The committee will also review states parties' progress in implementing the convention on a regular cycle (Articles 35-36) and report every two years to the UN General Assembly and the Economic and Social Council (Article 39).

It will be important for advocates to ensure that the committee's understanding of the convention is informed by an appreciation of the links between HIV and (other) disabilities. The committee should, for example, develop a "general recommendation" that outlines the application of the convention's provisions in the specific context of HIV/AIDS, making it clear that the convention applies to HIV/AIDS. Similarly, it will be important that the committee, in reviewing states parties' compliance with the convention, be aware of the ways in which the rights of PLHIV are infringed or unfulfilled. PLHIV groups and other AIDS advocates should take advantage of the review process to ensure that the committee receives relevant information about states under review and is equipped and encouraged to raise these HIV/AIDS-related concerns with states parties.

Fourth, the states that have ratified the Disability Convention will meet regularly in a "Conference of States Parties" to consider any matters with regard to the implementation of the convention (Article 40) [60]. Such conferences provide an opportunity for civil society organizations, and for UN agencies and experts (e.g., OHCHR and UNAIDS), to highlight the links between HIV and disability, to ensure attention to the HIV/AIDS-relevant aspects of the convention and to highlight the need for the convention's implementation in the context of HIV/AIDS-related discrimination. (Our thanks to Steve Estey of Disabled Peoples' International for this observation at the International Policy Dialogue on HIV/AIDS and Disability in Ottawa, 11-13 March 2009, sponsored by Health Canada's International Affairs Directorate.)

Finally, under the Optional Protocol to the Disability Convention, there will be the opportunity *vis-à-vis *those states parties that ratify the protocol, to pursue complaints against states regarding deficiencies in implementation of convention obligations. Individuals and groups of individuals (e.g., non-governmental organizations) can file complaints with the Committee on the Rights of Persons with Disabilities. Civil society advocates will certainly be turning their efforts to such "litigation" under the Optional Protocol; advocates for the rights of PLHIV in relevant countries should be considering opportunities for using this mechanism to challenge HIV-related discrimination.

There is growing recognition of the many links between HIV/AIDS and disability, with implications for direct services, for national programmes and policies on both HIV and disability, and for domestic and international law. The advent of the Disability Convention, with its recognition that disability is "an evolving concept", offers an opportunity for advocates to ensure that the newest addition to the core international human rights treaty becomes an additional tool for strengthening a human rights-based response to HIV/AIDS, both by advancing the rights of people whose pre-existing disability enhances their vulnerability to HIV infection and impedes their access to HIV/AIDS care, and by advancing the rights of people who are living with HIV/AIDS and experience the varying degrees to which the disease and its social reception are disabling. Making common cause between AIDS advocates and disability rights advocates, while being mindful of the similarities and differences in the ways in which HIV infection and other disabilities are experienced and perceived, will strengthen this common struggle for human rights.

## Competing interests

The authors declare that they have no competing interests.

## Authors' contributions

RE undertook research relevant to and drafted or contributed to all parts of the manuscript, as well as revising and editing the entire manuscript. LU undertook research relevant to and drafted or contributed to all parts of the manuscript. EZ contributed research and analysis to the discussion of the links between disability and HIV, and reviewed and commented on the entire manuscript. All the authors were involved in conceiving of the study and read and approved the final manuscript.

## Authors' information

RE is the executive director of the Canadian HIV/AIDS Legal Network. LU is a senior policy analyst with the Canadian HIV/AIDS Legal Network. EZ is the executive director of the Canadian Working Group on HIV and Rehabilitation.
